# The Effect of Butter Oil on Avoidance Memory in Normal and Diabetic Rats

**Published:** 2012

**Authors:** Khadije Zare, Seyed Reza Fatemi Tabatabaei, Ali Shahriari, Ramezan Ali Jafari

**Affiliations:** 1 *Department of Basic Sciences, Faculty of Veterinary Medicine, Shahid Chamran University of Ahvaz, Ahvaz, Iran*; 2 *Department of Basic Sciences, Faculty of Veterinary Medicine, Shahid Chamran University of Ahvaz, Ahvaz, Iran*; 3 *Department of Clinical Sciences, Faculty of Veterinary Medicine, Shahid Chamran University of Ahvaz, Ahvaz, Iran*

**Keywords:** Avoidance memory, Butter oil, Cholesterol, Diabetes, Hippocampus

## Abstract

**Objective(s):**

Since diabetes mellitus is accompanied by cognitive impairment in diabetic patient and animal models and since lipids play important roles in neuronal membrane composition, structure and function; we intended to evaluate the effect of dietary butter oil on passive avoidance memory of streptoztosin (STZ)-induced diabetic rats in this study.

**Materials and Methods:**

Thirty six adult male rats were randomly allocated to four equal groups: normal (N) and diabetic control (D) groups with free access to regular rat diet; but the diet of normal butter oil (NB) and diabetic butter oil (DB) groups was supplemented with 10% butter oil. Diabetes in D and DB groups was induced by intravenous (i.v.) injection of 50 mg/kg.bw of STZ. Passive avoidance memory and cholesterol of brain and hippocampal tissues has been measured six weeks after diabetes confirmation.

**Results:**

Diabetes, especially in diabetic butter oil group decreased the abilities of learning and memory. The level of cholesterol in hippocampus was higher in NB (*P*< 0.05) and DB (*P*< 0.01) groups.

**Conclusion:**

We suggest consumption of butter oil may worsen cognitive impairment of diabetic animal. This may be related to the higher elevation of cholesterol in the hippocampus of diabetic animals.

## Introduction

Diabetes mellitus, a major endocrine disorder and a growing health problem in most countries, is now emerging as a deadly disease (1). Diabetes has important effects on carbohydrate and lipid metabolism (2). 

Impairments in learning, memory, problem solving, mental and motor speed are more common in type 1 diabetic patients than in the general population (3). Cognitive deficits (4) and poor performances in abstract reasoning and complex psychomotor functioning (5) occur in type 2 diabetes (4). Impaired spatial learning and memory occur in animal models of both types 1 and 2 of diabetes (6). In the hippocampus of streptozotocin (STZ) induced diabetic rats, long-term potentiation is impaired, whereas long-term depression is enhanced, indicating altered hippocampal synaptic plasticity, which is associated with deficits in spatial learning and memory (7). 

Dietary fatty acids may, under certain conditions, induce changes in neurophysiological, cognitive and other behavioral variables (8). Dietary supplementation by a particular ratio of a mixture of n-3/n-6 poly unsaturated fatty acids (PUFAs) exerts many beneficial effects, such as a reduced cholesterol level and an increased level of PUFAs in the neuronal membrane (9). It is suggested that high fat diet may decrease memory and learning ability by changing the brain fatty acid composition (10)

The precursors of brain PUFAs, linoleic acid (18:2 n-6) and α-linolenic acid (18:3 n-3), can not be synthesized *de novo* by mammals, and are therefore considered nutritionally essential fatty acids (11). 

The fatty acid composition of milk fat typically comprises 70% saturated fatty acids, 25% monounsaturated fatty acids, and 5% polyunsaturated fatty acids (12). Milk butter is rich in cholesterol (13), and greater consumption of abundant saturated milk fatty acids, myristic (14:0), palmitic (16:0), and lauric (12:0) increases concentrations of LDL-c, whereas greater consumption of unsaturated fatty acids has the reverse effect (14).

The blood brain barrier effectively blocks uptake of cholesterol from the circulation, and thus brain cholesterol is derived mostly from *de novo* synthesis (15). There are many controversies about the effects of cholesterol on learning and memory, which pointed to the beneficial (16, 17) or worsening (18-20) effects of cholesterol. 

We did not find any report about the effect of oils, especially butter oil on diabetic animal cognition and cholesterol content of brain and hippocampus, thus this study was conducted to compare the effect of butter oil on avoidance memory and cholesterol content of brain and hippocampus in normal and diabetic rats.

## Materials

***Animals and grouping***

Male Wistar rats (180–200 g), from laboratory animal breeding council of Jundishapur University of Ahvaz, were used in this study. The animals were maintained in a controlled environment under the standard conditions of temperature at 23±1 °C; alternating light/dark repeated every 12 hr with food and water available freely throughout the study. Diabetes was induced by i.v. injection of 50 mg/kg STZ,) Alexis biochemicals, Switzerland) dissolved in 0.1 M citrate buffer (pH= 4.5). Non-diabetic animals were injected with the same volume of citrate buffer, as diabetic rats. One week later blood glucose of all rats was measured by glucometer (Bionime Rightest GM 300, Switzerland); those with plasma glucose greater than 300 mg/kg were considered as diabetic. 

Thirty six rats were assigned to four experimental groups. Normal (N) and diabetic (D) groups had *ad libitum* access to a normal diet prepared manually according to the rat requirement to energy, protein, vitamins and minerals (Table 1). Ten percent butter oil (prepared by warming of butter and removal of the lower water) was added to the diet of normal butter (NB) and diabetic butter (DB) groups and its protein, vitamins and minerals were balanced according to the energy change (Table 1). 

**Table 1:** The energy, protein, fat and fiber content per 100g of the prepared diet

	Normal diet	Butter oil enriched diet
Energy (Kcal)	301	355.2
Protein (g)	17.70	19.5
Fat (g)	5	14.05
Fiber (g)	5.6	5.2

Six weeks later, avoidance memory was tested. 

All rats were sacrificed at seventh week, their brain were removed immediately and kept at -20 ºC until assay of cholesterol and protein. The cholesterol and protein were measured in the brain and hippocampus of five rats in each group.

***Avoidance memory***

The experimental device was a 30 cm×30 cm with 50 cm height electronic avoidance-response chamber, made of plexiglas. The chamber has a bottom of parallel 2 mm stainless steel bars spaced 0.5 cm apart. A platform (5 cm high, 7 cm in diameter of its top surface) was fixedly placed at center on the bottom of the chamber, providing rats a shelter from the electronic attack. Before normal test, rats were continually trained in a one trial step-down inhibitory avoidance task 4 times (once a day, conducted between 10:00 and 12:00 a.m.), and tested for their memory retention of the escape platform from electronic attack at the same time 72 hr after training. Rats were placed on the platform, and their latency to step-down, first placing their four paws on the grids, was measured. In training sessions, immediately upon stepping down, the rats received a 0.5 mA, 2 sec foot shock. No foot shock was given in test sessions (21). Step down latencies and errors (during 2 min) were taken as a measure of memory retention.

***Biochemical parameters***

All the chemical materials for biochemical analysis were obtained from Merck chemicals Ltd, Germany). After removing right and left hippocampus from brain, lipids were extracted by Folch method (22). Briefly 2 ml of chloroform: methanol (2:1 V/V) was added to a test tube containing 0.1 g powdered tissue and five glass beads. Mixture was shaken overnight, and then centrifuged at 1000 g for 15 min, and the supernatant was removed, mixed with 1 ml of 0.9% NaCl solution and vigorously mixed for 1 min. The solution was centrifuged and the upper phase was discarded and the lower phase was removed, dried and reconstituted in 1 ml hexan-isopropanol (2:3 V/V) for lipid analysis. Cell derbies (sediment) was dried at room temperatre, dissolved in 0.1 N NaOH and used for total cell protein assay. Cholesterol was measured by oxidation to red polyene complex in presence of H_2_SO_4_ (23). Total protein was measured based on Bradford method, formation of blue complex between coomassie blue G-250 and protein (24).

***Statistics***

The data were analyzed by SPSS 16, showed as mean ± SEM and compared by one way ANOVA analysis by Duncan test rate of difference in each group and *P* values of less than 0.05 was considered to be significant. 

## Results

**Figure 1:**
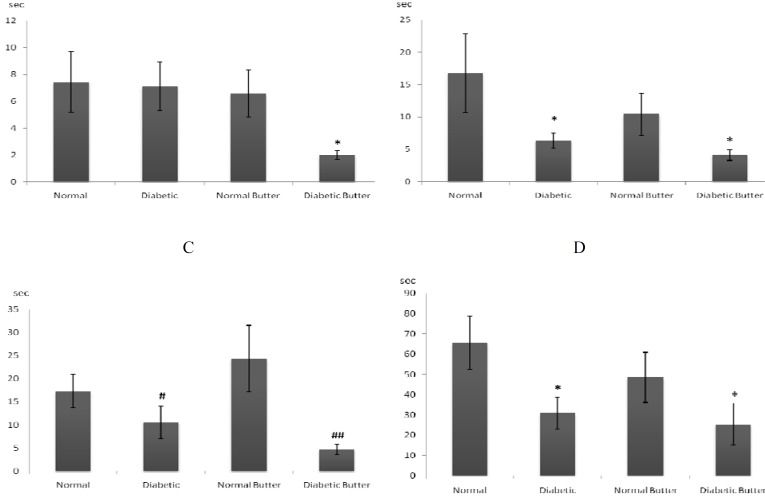
Effects of diabetes and butter oil on step down latency. A, B, C, and D: second, third, fourth and test session, respectively. n= 8 rats in each group. * indicate significant difference with N group (*P*< 0.05), # and ## indicate significant difference with normal butter (NB) group (*P*< 0.05 and *P*< 0.01)

There was no significant difference between groups at the first training session but at the second training session (Figure 1A) step down latency of DB group (2±0.33 sec) was shorter than the normal control (N) group (7.44± 2.24 sec) (*P*< 0.05), but there was no differences between the latency time of D and B groups (7.1±1.82 sec and 6.56±1.75 sec, respectively) with other groups.

At the third training session (Figure 1B) step down latency of D and DB groups (6.36±1.14 sec and 4.11±0.81 sec, respectively) was shorter than N group (16.78±6.12 sec) (*P*< 0.05), but there was no significant difference between NB (10.44± 3.28 sec) and N groups.

At the last training session (Figure 1C) time latency of D and DB (10.6±3.57 sec and 4.67±1.10 sec) groups was significantly (*P*< 0.05 and 0.01, respectively) shorter than NB group (24.33±7.22 sec) but there was no significant difference between N (17.33 ± 3.66 sec) and other groups.

At the test session (Figure 1D) step down latency of D and DB groups (30.9±7.63 sec and 25.33±10.44 sec, *P*< 0.05) was shorter than N group (65.56±13.19 sec) but there was no significant differences with NB group (48.56±12.27).

As showed in Table 2 brain cholesterol was not significantly different in any group, but the 

hippocampus content of cholesterol was significantly greater in B group in comparison with N group (*P*< 0.05), and in DB group in comparison with D and N groups *(P*< 0.05 and *P*< 0.01, respectively). 

The brain weight decreased by diabetes (*P*< 0.05), and addition of butter oil to the diet had no effect on it (Table 2).

**Table 2:** Effects of butter oil supplemented diet on cholesterol level of brain and hippocampus of normal and diabetic rats

Group	Brain (mg/g protein)	Hippocampus (mg/g protein)	Brain weight (g)
Normal control (n= 5)	17.40 ± 1.37	22.07 ± 2.47	1.860 ± 0.106
Diabetic control (n= 5)	17.55 ± 1.68	33.10 ± 6.24	1.730 ± 0.086*
Normal butter (n= 5)	17.51 ± 1.78	45.61 ± 8.13*	1.845 ± 0.120
Diabetic butter (n= 5)	21.34 ± 0.79	64.22 ± 10.09**#	1.721 ± 0.164*

* and ** significant difference with normal control group (*P*< 0.05 and *P*< 0.01), # significant difference with diabetic control group (*P*< 0.05)

## Discussion

According to the data, learning and memory retention were decreased by diabetes and they were worsened by supplementation of diet with butter oil. However, learning and memory behavior was similar in both normal groups (Figure 1A-C), thus consumption of butter oil in normal rats did not induce significant effect on learning and memory.

Avoidance memory task of diabetic rats have improved in some studies, related to more severe effect of electrical shock in diabetic animals (25), although its reduction in avoidance memory tasks have been shown in some studies (26, 27), however, in more complex task, such as Morris water maze and T-maze, diabetes were accompanied by a reduction in diabetic animals performance (24). In animal models of type I diabetes, such as that induced by STZ, reduced synaptic plasticity and impaired performances on behavioral learning tasks are common (28, 29). 

Cholesterol content of butter is high, and most of its fatty acids are saturated, with considerable amount of short chain fatty acids (12, 13). Moazedi *et al* (2002) showed that administration of 10% dietary butter in diet for 2 weeks increase spatial learning (*P*< 0.05) compared to control male rats. They conclude that the facilitative effects of dietary butter prior to training may be related to the presence of cholesterol or saturated fatty acids in butter (30). 

In the present study decreased memory of control diabetic and diabetic butter groups was along with the increase of cholesterol in hippocampus. Although dietary cholesterol does not cross the blood brain barrier (BBB) but there are a range of consequences of increasing cholesterol including significant peripheral pathology that may signal the brain along with a number of different pathways including cholesterol metabolites, pro-inflammatory mediators and antioxidant processes (31). It has been argued that the effect of oils on brain cholesterol may be resulted from the influence of the oils on *de novo* synthesis (32). Thus diabetes and/or butter oil supplemented diet may have activated mechanisms leading to hippocampus cholesterol elevation. Hyperglycemia, diabetes and high fat/high caloric diets which lead to hyperlipidemic state increase the free radical generation and oxidative stress (19, 33). Oxidative stress increases the cholesterol level in brain (34). These are some suggested reasons for cholesterol elevation in hippocampus of butter oil supplemented groups. On the other hand it has been reported that hypercholesterolemia increases the levels of reactive oxygen species so that it is possible that hypercholesterolemia facilitates the development of the neurodegenerative disease through increased oxidant production (35).

The importance of cholesterol for brain function is attested by the fact that brain itself has >2% cholesterol by weight. However, the mechanism by which cholesterol affects memory is unknown (16). Cholesterol is crucial for synapse generation, since it increases the number of synaptic vesicles, which contain high levels of cholesterol (36). Additionally, cholesterol is considered to be essential for remodeling neuronal membranes and growing new terminals, either during synaptic plasticity or in response to a neurodegenerative insult (37). 

Manipulations of cholesterol in animals have shown a number of different relationships between cholesterol and memory. Decreasing cholesterol in frontal cortex of rats by a 1:4 mixture of a-linolenic and linoleic acid improved learning and memory for tasks such as the water maze (38). Feeding mice a 2% cholesterol diet for eight weeks may result in deficits in working memory in the water Maze (20) but not always (39). Dietary cholesterol can influence a diverse number of learning tasks from water Maze to eyelid and fear conditioning even though cholesterol added to the diet does not cross the BBB. The human cholesterol literature is no less complex. Correlations of cholesterol levels with cognitive function have been found to be positive, negative, or to have no relationship at all (31).

There is an inverse relationship between alfa-linolenic acid and cholesterol level (40). Although there is a little n-3 and n-6 fatty acids in dietary butter (12), it may be lower than to reduce the cholesterol level of the hippocampus in the recent study.

A key function of cholesterol is to regulate membrane fluidity. By decreasing membrane fluidity, cholesterol affects the biophysical properties of the membrane, thereby affecting the functioning of membrane-bound proteins such as ion channels and receptors, and alterations in cholesterol levels of rat hippocampal neurons have been shown to affect their excitability (41). 

## Conclusions

According to this study, it is concluded that diabetes has detrimental effects on cognition and consumption of butter oil as an animal source fat, with a little PUFA, worsens it, thus it may be a predisposing factor in improvement of Alzheimer disease. These effects may be related to the elevation of cholesterol in hippocampus of diabetic rats, especially followed butter oil consumption in diabetic rats and the differences between normal and diabetic animal metabolism of brain following insulin deficiency and/or oxidative stress.
